# Understanding Barriers to Cervical Cancer Screening in Women With Access to Care, Behavioral Risk Factor Surveillance System, 2014

**DOI:** 10.5888/pcd13.160225

**Published:** 2016-11-10

**Authors:** Anatasha Crawford, Vicki Benard, Jessica King, Cheryll C. Thomas

**Affiliations:** Author Affiliations: Vicki Benard, Jessica King, Cheryll C. Thomas, Centers for Disease Control and Prevention, Division of Cancer Prevention and Control, Atlanta, Georgia.

## Abstract

Cervical cancer screening can save lives when abnormal cervical lesions and early cancers are detected and treated; however, many women are not screened as recommended. We used the Behavioral Risk Factor Surveillance System survey to examine nonfinancial barriers to cervical cancer screening among women who reported having insurance and a personal doctor or health care provider. Among these women, a higher proportion who were never or rarely screened reported having multiple chronic conditions. The results of this study underscore the importance of incorporating preventive clinical services into the management of one or more chronic conditions.

## Objective

Widespread use of the Papanicolaou (Pap) test has decreased cervical cancer incidence and deaths. Over half of all new cervical cancers are estimated to occur in women who have never or rarely been screened (1). Limited or no access to health care is a known barrier to screening (2). In a 2012 study of women with no cervical cancer screening in the past 5 years, nearly 70% had health insurance and a regular doctor or health care provider (3). This analysis examines nonfinancial barriers to meeting cervical screening recommendations, focusing on women aged 40 to 65 years who may be seeking other preventive screening (eg, mammogram, colonoscopy) (4).

## Methods

The Behavioral Risk Factor Surveillance System (BRFSS) survey is a state-based, random-digit–dialed telephone survey of the civilian, noninstitutionalized adult population of the United States (5). Survey data were available for all 50 states and the District of Columbia in 2014.

Women were asked if they had ever had a Pap test and if so, when this test was last performed. We selected women who were aged 40 to 65 years, reported having medical insurance and at least one personal doctor or health care provider, had not had a hysterectomy, and were not pregnant at the time of the survey. In addition to screening with a Pap test alone every 3 years, current cervical cancer screening recommendations include the use of the human papillomavirus (HPV) test with the Pap test every 5 years (4). Because HPV testing rates could not be assessed for all 50 states and the District of Columbia, respondents were categorized as never or rarely screened if they reported never having a Pap test or not having one in more than 5 years to account for the possibility that a woman may have had an HPV test (women aged 30 to 65 years who want to lengthen the screening interval and be screened with a combination of Pap test and HPV test every 5 years). Comparisons were made with women who were up to date with screening (Pap test within 3 years). Respondents who refused to answer or answered “don't know or not sure” were excluded. Eleven chronic conditions (heart attack, heart disease, stroke, asthma, skin cancer, cancer other than skin, chronic obstructive pulmonary disease [COPD], arthritis, depression, kidney disease, and diabetes) were ascertained by BRFSS and analyzed for this study. BRFSS data were weighted by using advanced raking techniques (6) and were age-adjusted to the 2014 BRFSS population. We performed χ^2^ testing to compare the characteristics of the respondents across screening history (screened versus never or rarely screened).

## Results

All variables were significant (except “asthma now” and cancer other than skin”) when comparing screened versus never or rarely screened women (*P* < .05) (Table). Compared with women screened on time, a higher proportion of women never or rarely screened were aged 60 to 65 years (27.6%), Asian/Pacific Islander (7.0%), never married (16.2%), obese (37.3%), current smokers (25.6%), and had an annual income less than $10,000 (11%). A significantly higher proportion of preventive care measures (mammogram [83.2%], clinical breast examination [73.6%], and colorectal cancer screening [69.9%]) were observed among women who received timely cervical cancer screening when compared with women never or rarely screened (*P* = .001).

**Table:** Demographic Characteristics of US Women^a^ Aged 40–65 Years With Health Insurance and a Regular Health Care Provider (N = 66,402) Tested for Cervical Cancer by Papanicolaou (Pap) Test, BRFSS, 2014

Characteristic	Screened on Time^b^	Never or Rarely Screened^b^	*P* Value^d^
	N (%)^c^		
**Demographic Characteristic**			
**Age, y**			
Total	60,371 (100.0)	6,031 (100.0)	.001
40–44	9,095 (21.9)	577 (16.0)	
45–49	9,731 (17.9)	709 (14.1)	
50–54	12,138 (22.9)	1,122 (21.6)	
55–59	12,993 (17.8)	1,391 (20.7)	
60–65	16,414 (19.4)	2,232 (27.6)	
**Race/ethnicity**			
Total	59,618 (100.0)	5,938 (100.0)	.02
Non-Hispanic white	48,771 (71.4)	4,803 (71.4)	
Non-Hispanic black	4,963 (11.7)	413 (10.1)	
Asian/Pacific Islander	1,069 (4.6)	175 (7.0)	
American Indian/Alaskan Native	727 (0.8)	124 (1.2)	
Other	1,134 (1.4)	146 (1.6)	
Hispanic	2,954 (10.0)	277 (8.7)	
**Marital status**			
Total	60,017 (100.0)	5,983 (100.0)	.001
Married	38,834 (68.0)	2,878 (49.5)	
Divorced	9,581 (13.6)	1,174 (18.9)	
Widowed	3,473 (4.4)	655 (9.2)	
Separated	1,356 (2.7)	179 (3.8)	
Never married	5,622 (9.1)	983 (16.2)	
Member of an unmarried couple	1,151 (2.3)	114 (2.4)	
**Annual income, $**			
Total	53,727 (100.0)	5,247 (100.0)	.001
<10,000	1,837 (4.0)	461 (11.0)	
10,000–14,999	1,806 (3.6)	464 (10.2)	
15,000–19,999	2,269 (4.6)	471 (9.1)	
20,000–24,999	2,894 (5.4)	545 (10.0)	
25,000–34,999	4,068 (7.8)	633 (12.4)	
35,000–49,999	7,062 (12.6)	808 (12.9)	
50,000–74,999	9,887 (16.8)	758 (13.0)	
≥75,000	23,904 (45.3)	1,107 (21.4)	
**Education**			
Total	60,230 (100.0)	6,006 (100.0)	.001
Did not graduate high school	2,109 (7.7)	521 (15.0)	
Graduated high school	12,477 (22.3)	1,920 (33.0)	
Attended college/technical school	16,250 (32.3)	1,753 (31.6)	
Graduated college/technical school	29,394 (37.7)	1,812 (20.4)	
**Body mass index, kg/m^2^**			
Total	56,054 (100.0)	5,457 (100.0)	.001
Underweight, ≤18	845 (1.6)	133 (2.2)	
Normal, ≤25	21,645 (38.0)	1,670 (32.1)	
Overweight, >25 to <30	17,264 (30.5)	1,531 (28.3)	
Obese, ≥30	16,300 (29.9)	2,123 (37.3)	
**Residence**			
Total	42,245 (100.0)	4,274 (100.0)	.003
Metropolitan^e^	22,885 (65.5)	1,938 (59.2)	
Nonmetropolitan	19,360 (34.5)	2,336 (40.8)	
**Smoking status**			
Total	60,127 (100.0)	6,010 (100.0)	.001
Current smoker^f^	7,477 (12.8)	1,492 (25.6)	
Former smoker	14,790 (23.3)	1,386 (23.6)	
Never smoked	37,860 (63.9)	3,132 (50.8)	
**Use of Preventive Care**			
**Breast cancer screening**			
Total	60,200 (100.0)	5,980 (100.0)	.001
Yes g	50,717 (83.2)	1,895 (34.5)	
No	9,483 (16.8)	4,085 (65.5)	
**Clinical breast examination**			
Total	60,037 (100.0)	5,913 (100.0)	.001
In last year	44,531 (73.6)	1,159 (22.0)	
>1 Year ago	14,159 (23.4)	3,627 (56.5)	
Never	1,347 (3.0)	1,127 (21.5)	
**Colorectal cancer screening, aged 50–65 y^h^**			
Total	40,214 (100.0)	4,558 (100.0)	.001
Yes	28,993 (69.9)	1,643 (34.5)	
No	11,221 (30.1)	2,915 (65.5)	
**Chronic Disease History**			
**Heart attack**			
Total	60,258 (100.0)	5,990 (100.0)	.001
Yes	1,216 (2.0)	316 (4.2)	
No	59,042 (98)	5,674 (95.8)	
**Heart disease**			
Total	60,173 (100.0)	5,979 (100.0)	.001
Yes	1,436 (2.4)	331 (4.9)	
No	58,737 (97.6)	5,648 (95.1)	
**Stroke**			
Total	60,259 (100.0)	6,011 (100.0)	.001
Yes	1,327 (2.1)	290 (4.5)	
No	58,932 (97.9)	5,721 (95.5)	
**Asthma**			
Total	60,225 (100.0)	6,016 (100.0)	.004
Yes	8,626 (14.3)	1,085 (18.1)	
No	51,599 (85.7)	4,931 (81.9)	
**Asthma now**			
Total	8,441 (100.0)	1,063 (100.0)	.26
Yes	6,468 (76.8)	851 (79.6)	
No	1,973 (23.2)	212 (20.4)	
**Chronic obstructive pulmonary disease**			
Total	60,191 (100.0)	6,004 (100.0)	.001
Yes	3,645 (6.0)	834 (13.7)	
No	56,546 (94.0)	5,170 (86.3)	
**Arthritis**			
Total	60,113 (100.0)	6,000 (100.0)	.001
Yes	19,462 (31.4)	2,405 (38.1)	
No	40,651 (68.6)	3,595 (61.9)	
**Depression**			
Total	60,147 (100.0)	6,001 (100.0)	.001
Yes	14,142 (22.0)	1,810 (31.4)	
No	46,005 (78.0)	4,191 (68.6)	
**Kidney disease**			
Total	60,251 (100.0)	6,002 (100.0)	.001
Yes	1,501 (2.5)	241 (3.8)	
No	58,750 (97.5)	5,761 (96.2)	
**Diabetes**			
Total	60,302 (100.0)	6,019 (100.0)	.001
Yes	5,652 (9.6)	1,011 (15.4)	
Only during pregnancy	1,061 (1.9)	99 (1.5)	
No	52,714 (86.8)	4,804 (81.4)	
Prediabetes	875 (1.7)	105 (1.7)	
**Skin cancer**			
Total	60,261 (100.0)	6,014 (100.0)	.004
Yes	4,095 (6.1)	288 (4.4)	
No	56,166 (93.9)	5,726 (95.6)	
**Cancer, nonskin**			
Total	60,251 (100.0)	6,003 (100.0)	.05
Yes	4,288 (6.6)	402 (5.4)	
No	55,963 (93.4)	5,601 (94.6)	

Abbreviation: BRFSS, Behavioral Risk Factor Surveillance System.

a Women who had not had a hysterectomy and are not currently pregnant.

b Adapted from the US Preventive Services Task Force recommendations for cervical cancer. Since human papilloma virus testing could not be assessed for all 50 states and the District of Columbia, “on time” is based on having had a Pap test within the past 3 years. “Never or rarely screened” is based on ever having had a Pap test in more than 5 years to account for the possibility that a woman may have had a human papilloma virus test. Data for women screened more than 3 years but less than 5 years ago is not shown.

c Percentages are age-adjusted to the 2014 BRFSS population.

d χ^2^ Testing used to determine association.

e Metropolitan is defined as the center city of a metropolitan statistical area or outside the center city of a metropolitan statistical area but does not include the county.

f Current smoker is defined as smoked at least 100 cigarettes in their lifetime and now smoke every day or some days.

g Mammogram within the past 2 years.

h Fecal occult blood test (FOBT) in 1 year, colonoscopy in 10 years or flexible sigmoidoscopy in 5 years and FOBT in 3 years.

Women who were never or rarely screened for cervical cancer had a higher prevalence of ever reporting 1 of 7 chronic conditions: heart disease (4.9%), COPD (13.7%), arthritis (38.1%), depression (31.4%), kidney disease (3.8%), or diabetes (15.4%) than women who were regularly screened (*P* < .01). Higher proportions of never or rarely screened women also reported having had a heart attack (4.2%) or a stroke (4.5%) in their lifetime than regularly screened women. Women with skin cancer were more likely to be screened for cervical cancer (6.1%, *P* = .004); however, there was no significant difference (*P* = .05) in the proportions of women screened for cervical cancer who had had other forms of cancer. A significantly higher proportion of women (*P* = .001) who were never or rarely screened had more than 1 or 2 chronic conditions (3 or 4 chronic conditions, 16.6%; 5 or more chronic conditions, 3.8%) (Figure). Women screened every 3 years were more likely to have no chronic conditions (48.0%, *P* = .001).

**Figure:**
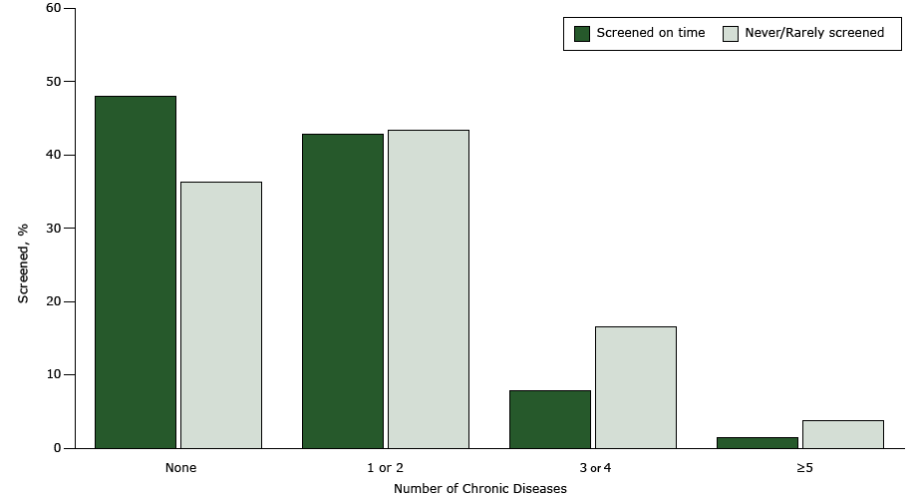
Percentage of women aged 40 to 65 years with health insurance and a regular health care provider screened for cervical cancer by Papanicolaou (Pap) test, by number of chronic diseases, BRFSS, 2014. Chronic diseases analyzed were heart attack, heart disease, stroke, asthma, chronic obstructive pulmonary disease, arthritis, depression, kidney disease, diabetes, skin cancer, and cancer other than skin. Women who had had a hysterectomy or were pregnant at the time of the survey were excluded. Data were age-adjusted to the 2014 BRFSS population. Screening (once every 3 years) is based on the US Preventive Services Task Force recommendations for cervical cancer screening. Since HPV testing could not be assessed for all 50 states and the District of Columbia, “on time” is based on having had a Pap test within the past 3 years. Never or rarely screened refers to women who ever had a Pap test in more than 5 years to account for the possibility that a woman may have had an HPV test (women aged 30 to 65 years who want to lengthen the screening interval can be screened with a combination of Pap test and HPV test every 5 years). Data for women screened more than 3 years ago but less than 5 years ago are not shown. Abbreviation: BRFSS, Behavioral Risk Factor Surveillance System; HPV, human papilloma virus. **Table:** 

No. of Chronic Diseases	%
**Screened on time**	
None	48.0
1 or 2	42.8
3 or 4	7.9
≥5	1.4
**Never/rarely screened**	
None	36.3
1 or 2	43.4
3 or 4	16.6
≥5	3.8

## Discussion

When we examined factors related to cervical cancer screening among women who reported having health insurance and access to a regular doctor or health care provider, a larger proportion of women with multiple chronic conditions reported not receiving the recommended screening for cervical cancer. Our findings were similar to others indicating that insured women with arthritis, diabetes, and myocardial infarction were less likely to be screened for cervical cancer (7–10). In addition, we found that a larger proportion of women with COPD, depression, heart disease, or kidney disease did not adhere to cervical cancer screening recommendations compared with women without these conditions. Studies of women with chronic conditions who received regular health care from primary care physicians and specialists showed similar findings when examining breast and colorectal cancer screening (8,11).

Limitations of this study are not examining younger women who were eligible for screening and using self-reported survey data. Previous studies suggest that age, lack of awareness, lack of transportation, fatalistic health beliefs, low health literacy, poor patient compliance to provider recommendations, and low-quality health care services may prevent insured women with chronic conditions from obtaining timely cervical cancer screening (3,7,12). The primary reason women adhere to timely cancer screening is because of encouragement from a provider; however, disease management for women with multiple chronic conditions is given greater priority than disease prevention (7,9). Additional research is recommended to determine if physicians can effectively balance managing patients’ chronic conditions and ensuring that patients receive recommended preventive care services. 
